# Genome-wide scan reveals population stratification and footprints of recent selection in Nelore cattle

**DOI:** 10.1186/s12711-018-0381-2

**Published:** 2018-05-02

**Authors:** Diercles F. Cardoso, Lucia Galvão de Albuquerque, Christian Reimer, Saber Qanbari, Malena Erbe, André V. do Nascimento, Guilherme C. Venturini, Daiane C. Becker Scalez, Fernando Baldi, Gregório M. Ferreira de Camargo, Maria E. Zerlotti Mercadante, Joslaine N. do Santos Gonçalves Cyrillo, Henner Simianer, Humberto Tonhati

**Affiliations:** 1 Department of Animal Science, Faculty of Agrarian and Veterinary Sciences, Sao Paulo State University, Jaboticabal, SP Brazil; 2 National Counsel of Technological and Scientific Development (CNPq), Brasília, DF Brazil; 3 Animal Breeding and Genetics Group, Department of Animal Sciences, University of Goettingen, Goettingen, Germany; 4 Present Address: Institute for Animal Breeding, Bavarian State Research Center for Agriculture, Grub, Germany; 5 APTA Beef Cattle Center, Institute of Animal Science, Sertãozinho, SP Brazil

## Abstract

**Background:**

This study aimed at (1) assessing the genomic stratification of experimental lines of Nelore cattle that have experienced different selection regimes for growth traits, and (2) identifying genomic regions that have undergone recent selection. We used a sample of 763 animals genotyped with the Illumina BovineHD BeadChip, among which 674 animals originated from two lines that are maintained under directional selection for increased yearling body weight and 89 animals from a control line that is maintained under stabilizing selection.

**Results:**

Multidimensional analysis of the genomic dissimilarity matrix and admixture analysis revealed a substantial level of population stratification between the directional selection lines and the stabilizing selection control line. Two of the three tests used to detect selection signatures (*F*_ST_, XP-EHH and iHS) revealed six candidate regions with indications of selection, which strongly indicates truly positive signals. The set of identified candidate genes included several genes with roles that are functionally related to growth metabolism, such as *COL14A1*, *CPT1C*, *CRH*, *TBC1D1*, and *XKR4*.

**Conclusions:**

The current study identified genetic stratification that resulted from almost four decades of divergent selection in an experimental Nelore population, and highlighted autosomal genomic regions that present patterns of recent selection. Our findings provide a basis for a better understanding of the metabolic mechanism that underlies the growth traits, which are modified by selection for yearling body weight.

**Electronic supplementary material:**

The online version of this article (10.1186/s12711-018-0381-2) contains supplementary material, which is available to authorized users.

## Background

Human/animal interactions in livestock production have led to major phenotypic changes in animal traits, which are mainly due to the effect of selection [1]. Anthropogenic selection, along with natural selection and adaptation, have increased the frequency of favorable alleles and resulted in changes in the frequencies of their surrounding loci [2], thereby establishing genomic signatures of positive selection. In turn, the methods used to identify this type of signature of selection represent powerful tools to detect evidence of selection during the process of animal domestication or breed formation, and ultimately they aid in elucidating the mechanisms that underlie the morphological, productive and functional traits of a species [3].

Cattle are domestic animals used worldwide, with two divergent groups: taurine (*Bos primigenious taurus*) and indicine (*Bos primigenious indicus*) cattle, each of which includes several breeds, which have been established for different purposes by humans. One common strategy for investigating past selection in cattle involves the systematic comparison of the genomes of breeds that have evolved under different selection regimes, e.g., dairy versus beef breeds [4, 5]. The motivation behind such comparisons is to identify strongly differentiated genomic regions between different groups of animals. However, analysis of within-breed stratification may allow the identification of signatures of selection within a single breed for which genetically distinct lines have been established. One example of such a study in cattle involved Holstein lines that were divergently selected for milk yield, in which the identified signatures of selection co-localized with known quantitative trait loci (QTL) that affect milk traits [6, 7].

Nelore cattle (originally named Ongole in India) are an indicine breed, which is predominantly raised in Brazil for meat production. Apart from the national Nelore breeding programs, there is an experimental program with three selection lines that has been running since 1978 at the APTA Beef Cattle Center—Institute of Animal Science (IZ), Sertãozinho—Brazil. This program was initiated to demonstrate to producers the benefits of selection for growth traits and its economic impact on the bovine industry [8]. Briefly, three selection lines were established in 1980 by dividing an experimental herd representative of the main Nelore lineages that currently exist in Brazil. Since then, a line referred to as Nelore Control (NeC) has been maintained under stabilizing selection, in which animals with a yearling body weight (YBW) close to the average of the contemporary group are annually selected for reproduction. The two other lines, Nelore Selection (NeS) and Nelore Traditional (NeT), have independently undergone selection for increased YBW. While the NeS and NeC lines are maintained as closed lines, the NeT line has received sires from the two other lines (7 from NeC and 47 from NeT) and commercial herds.

After almost 40 years of selection, distinct phenotypic differences are observed between the lines undergoing stabilizing and directional selection, including differences in average body weight at various ages, body measurements, scrotal circumference and carcass quality [8–10]. Inbreeding level in these lines is controlled by adopting breeding strategies such as exclusion of siblings as sires, short mating time for each selected sire (only two seasons), and avoidance of mating between closely-related animals. In 2003, the observed coefficients of inbreeding in the three lines ranged from 4 to 4.3% [11], while in animals born in 2012 they ranged from 4.2 to 6.4%.

The existence of these experimental bovine lines has instigated studies on candidate genes, with the goal of detecting polymorphisms that show extreme differences in allele frequencies between lines, which may be linked to phenotypic differences [12–14]. However, genome-wide screening of high-density single nucleotide polymorphism (SNP) panels to detect selection is probably more suitable for revealing genomic regions that have undergone selection in this population and should help to understand the genetic mechanisms that underlie response to selection. Thus, the aims of this study were (1) to assess the effect of selection within this experimental Nelore population and (2) to scan for signatures of selection left by recent directional selection.

## Methods

### Description of the experimental selection program

Since the experimental program was established in 1980, the NeC, NeS and NeT lines are composed of 60, 120 and 170 cows with four, six and up to eight bulls, respectively, with 50% of the bulls at 2 years of age (first mating) and 50% at 3 years (second and last mating). The females that were initially assigned to each line were randomly chosen from an experimental herd of 350 cows, and since then, the annual culling rate of cows is approximately 20%, with replacement females for each line being selected based on their YBW at 550 days of age and raised on pasture. The bulls that were initially assigned to each line were chosen based on their YBW at 378 days of age, after 168-day feedlot performance tests. The bulls that were assigned to the NeC line had a YBW close to the average value for bulls with the same birth year (1977 or 1978), while those assigned to the NeS and NeT lines had a higher YBW. This bull selection scheme was maintained to the present day, by replacing annually the 50% older bulls within each line. Average annual selection intensities of 0.05 ± 0.24, 0.42 ± 0.29 and 0.56 ± 0.28 were applied to NeC, NeS and NeT females, respectively, while the corresponding values for males are equal to − 0.03 ± 0.20, 1.46 ± 0.30 and 1.60 ± 0.54.

**Fig. 1:**
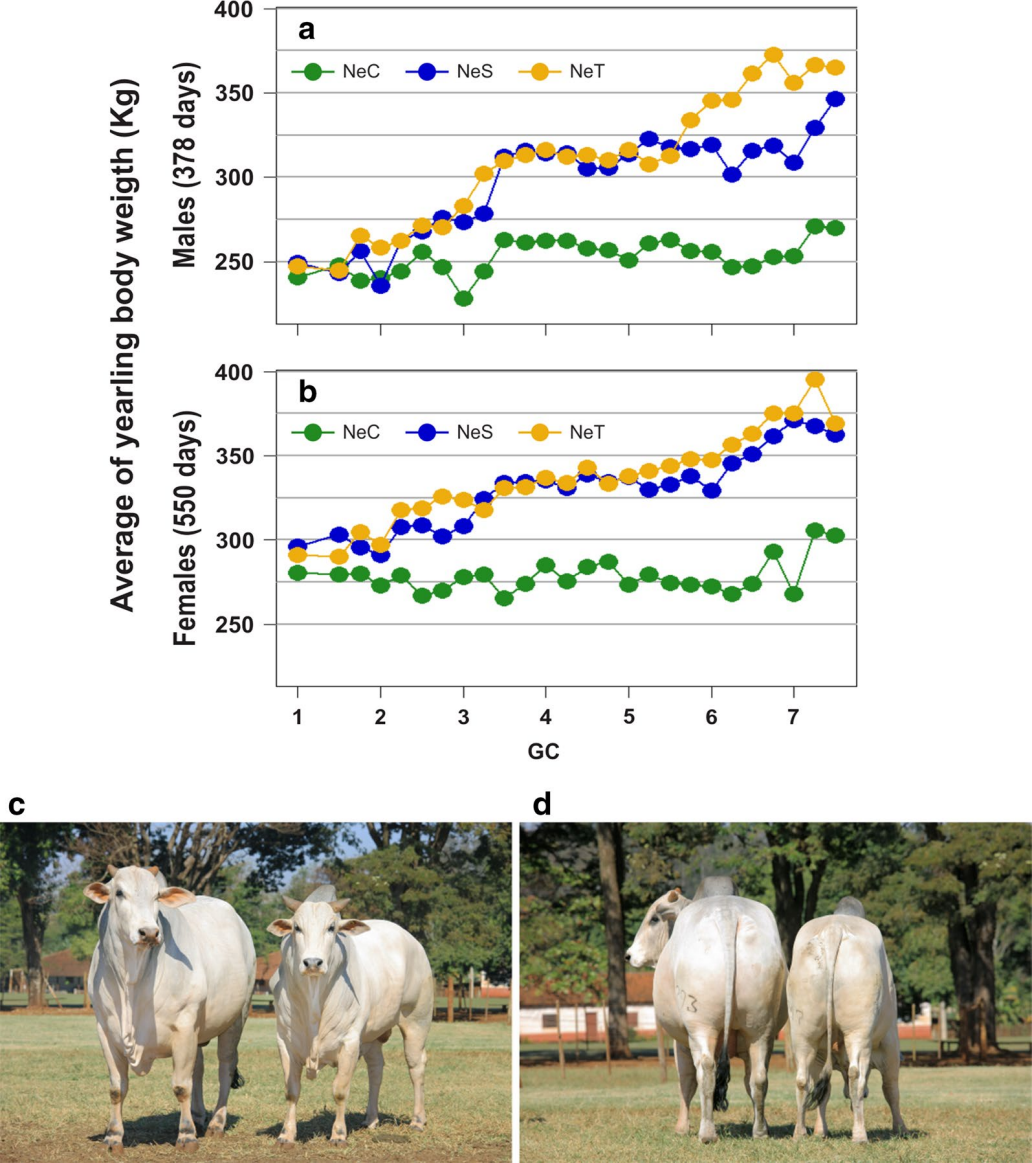
Direct responses to selection for yearling body weight across generations. **a** and **b** Average yearling body weights (YBW) in Nelore Control (NeC), Nelore Selection (NeS) and Nelore Traditional (NeT) lines, within generation coefficient (GC) classes with intervals of 0.25, **c** and **d** 4 year old sires from the NeS (left) and NeC (right) lines

Figure 1 shows the phenotypic differentiation in YBW between the lines obtained via selection. The individuals’ generation coefficients (GC) were determined by the method described by Brinks et al. [15], where the GC of each calf was the average of its parents’ GC values plus 1. The founder animals used for establishing the experimental lines were assigned a GC of 0.

### Sampling of animals and quality control procedures

We used a subsample of 782 animals born between 2004 and 2012 (92 NeC, 192 NeS and 498 NeT) that were previously genotyped because of phenotypes related to feed efficiency [16, 17]. The average GC for this subsample was equal to 6.1 (ranging from 4.4 to 7.7) and generation 6 had the broadest representation of animals from each line [see Additional file 1: Table S1]. The average estimated inbreeding coefficients in the genotyped subsample were equal to 0.05 ± 0.02 for NeC (ranging from 0.02 to 0.18), 0.03 ± 0.01 for NeS (ranging from 0.01 to 0.06) and 0.03 ± 0.01 for NeT (ranging from 0.01 to 0.08). Table 1 presents the average additive relationships between the genotyped animals of the three lines, which were estimated based on the full pedigree (see Silva et al. [17] for a complete pedigree description).

**Table 1:** Average additive genetic relationships in the genotyped subsample

	NeC	NeS	NeT
NeC	0.180 ± 0.072	0.007 ± 0.001	0.007 ± 0.001
NeS		0.115 ± 0.060	0.053 ± 0.035
NeT			0.091 ± 0.059

Each animal was genotyped using the Illumina^®^ BovineHD BeadChip (San Diego, CA). Quality control (QC) procedures were performed using Plink (version 1.07) [18]: samples with call rates lower than 0.90 were removed, and SNPs with call rates lower than 0.95, non-autosomal, unmapped, and duplicated SNPs or those that were monomorphic in all lines were also discarded. In addition, SNPs that might be misplaced were removed based on the decay of r^2^ as a function of physical distance. Following an algorithm adapted from Bohmanova et al. [19], this QC procedure consisted of: (1) recording the distance between each SNP and the top 10 syntenic SNPs with the highest r^2^ values; (2) flagging a particular SNP once for each distance greater than 10 Mb within the list of its top 10 r^2^ and compiling a list of candidate misplaced SNPs for each chromosome; (3) excluding from further analysis the SNPs with the highest flag counts on each chromosome; and (4) repeating steps (1), (2) and (3) with the list of candidate misplaced SNPs, until the list is empty (an example of the removal of a misplaced SNP is in Figure S1 [see Additional file 2: Figure S1]).

Finally, 529,172 SNPs remained for further analyses [see Additional file 1: Table S2]. Next, imputation of missing genotypes and haplotype phasing were performed by combining all the samples together, regardless of their respective line, using Beagle software (version 3.3.2) [20].

### Population structure analyses

Two different methods were used to assess population stratification. First, a multidimensional scaling (MDS) analysis was applied using the genomic distance matrix and the “cmdscale” function from the “stats” package in R software [21]. The genomic distance between two individuals was estimated as 1 minus the proportion of identical by state (IBS) alleles that they share. Second, an admixture analysis was performed to measure the proportion of individual ancestry from different numbers of hypothetical ancestral populations, using the ADMIXTURE software (version 1.04) [22]. Thus, an unsupervised model-based approach that relies on maximum likelihood was applied to the genotype matrix, using hypothetical ancestral population (K) numbers of 2 and 3, which reflect the different selection regimes and the number of lines in the subsample used for this study, respectively.

### Wright’s fixation index (*F*_ST_)

Wright’s fixation index (*F*_ST_) was computed for each pairwise combination of lines based on the estimator corrected for unequal sample sizes [23] implemented in the “fsthet” package [24] in R software [21], using all SNPs that had passed the QC procedures. Then, *F*_ST_ and heterozygosity averages were estimated for genomic windows of 100 kb across the autosomes, with an overlap of 75 kb. Putative signals of divergence were detected with two empirical strategies that are typically used in studies involving *F*_ST_ tests, one that identifies the top-ranking windows [25, 26] and another method that identifies outliers from the background level of *F*_ST_-heterozygosity [24, 27]. Thus, windows were considered to show putative signatures of selection if they contained more than five SNPs, presented an average *F*_ST_ within the top 1%, and outside the 95% confidence interval of their heterozygosity bin in both cross-line comparisons of NeC with one of the lines under directional selection (“NeC vs. NeS” and “NeC vs. NeT”) [see Additional file 2: Figure S2].

### Haplotype-based methods for assigning signatures of selection

The extended haplotype homozygosity (EHH) metric is defined as the probability of randomly choosing two identical extended haplotypes that surround a specific locus [28], for which higher values are expected for selected alleles with a frequency that increased rapidly due to selection. In our study, we applied two somewhat complementary EHH-derived methods to detect signatures of selection, the cross population EHH (XP-EHH) [29] and the integrated haplotype homozygosity score (iHS) [30], both of which were estimated using the SELSCAN software (version 1.1.0) [31]. Whereas XP-EHH is a cross population method that is useful to detect signals of divergent selection between lines, iHS is a within-population method that allows the identification of signals of ongoing positive selection.

The XP-EHH test was applied to the comparisons between NeC and each of the lines under directional selection (“NeC vs. NeS” and “NeC vs. NeT”). XP-EHH values were averaged for 100-kb windows with an overlap of 75 kb, and a cut-off threshold of 2.58 (*p* < 0.005) was applied to determine candidate signatures of selection. Only windows for which the average XP-EHH exceeded the upper cut-off in both cross-line comparisons and contained more than five SNPs were considered to harbor a signature of selection [see Additional file 2: Figure S3], which was characterized by the presence of some high-frequency haplotypes in the NeS and NeT lines but not in the NeC line.

The iHS test was performed by considering the lines under directional selection (NeS and NeT) together after removing SNPs with a minor allele frequency (MAF) lower than 0.01, since iHS is a within-population methodology that is not appropriate for analyzing SNPs close to fixation. One allele of each SNP was arbitrarily assigned as the “reference” allele, and absolute values of iHS (|iHS|) were then used to identify signatures of selection, as in Cohen-Zinder et al. [32]. The “reference” allele was randomly determined based on the high similarity observed between iHS values obtained from a subset of SNPs, with or without ancestral allele information retrieved from two cattle reference allele lists [33, 34] [see Additional file 2: Figure S4]. The |iHS| values of 494,372 SNPs were divided into 20 bins based on the frequency of the assigned ancestral allele, and the values were then standardized within each bin. The |iHS| values were averaged across 100-kb windows, with an overlap of 75 kb. Windows with |iHS| scores higher than 2.58 (*p* < 0.005) were considered as putative signatures of selection.

### QTL and gene annotation

Known QTL that overlapped with each detected signature of selection were retrieved from the CattleQTL database [35]. Genes were identified within each putative selected region using the Ensembl cow gene set 84 [36]. Enrichment of genes in specific biological processes and molecular pathways was then analyzed using the DAVID database (version 6.7) [37], however since no significant pathway was detected, the results are not shown.

## Results and discussion

### Polymorphisms and population structure

After QC, the genotyped population was composed of 89, 189 and 485 samples from NeC, NeS, and NeT lines, respectively [see Additional file 1: Table S1]. The NeC line presented the largest number of monomorphic SNPs and the lowest heterozygosity (Table 2), which may be due to its smaller effective population size (N_e_) relative to the population sizes of the other two lines. Current N_e_ values of 51, 88 and 177 were estimated for the NeC, NeS and NeT lines, respectively, based on the increase in individual inbreeding since the establishment of these lines, according to Gutiérrez et al. [38]. However, directional selection in successive generations, as experienced by the NeS and NeT lines, can result in the loss of rare alleles and an increase in apparent inbreeding, which may explain why these three lines that differ significantly in effective population size, present similar proportions of SNPs with a low MAF [see Additional file 2: Figure S5].

**Table 2:** Summary statistics for SNPs in the three selection lines

Line	Monomorphic SNPs (N)	Heterozygosity ± SD
NeC	74,896	0.26 ± 0.01
NeS	26,923	0.28 ± 0.01
NeT	9456	0.28 ± 0.01

*NeC* Nelore control, *NeS* Nelore selection; *NeT* Nelore traditional

**Fig. 2:**
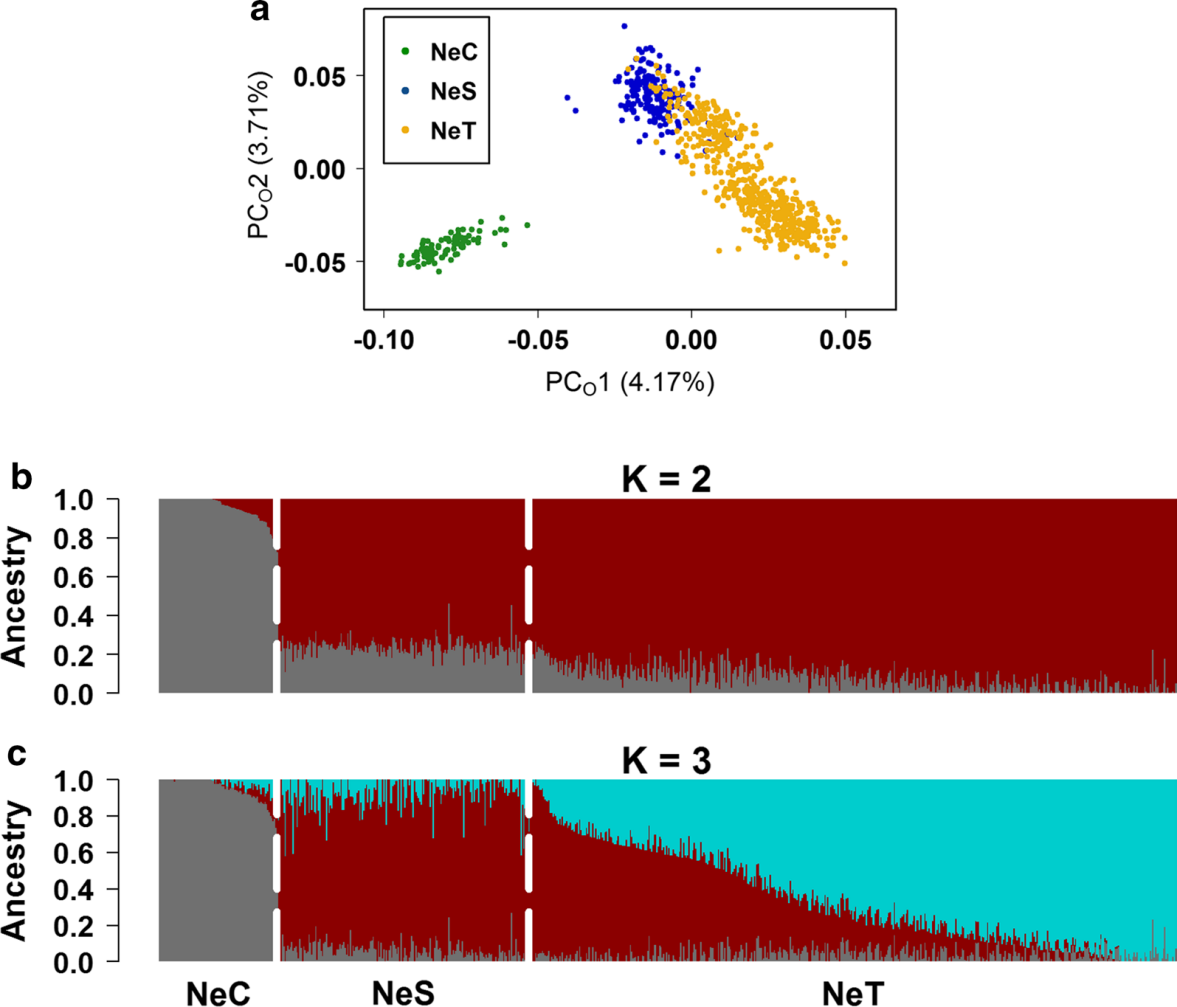
Population structure, as revealed by MDS and admixture analyses. **a** Depicts the distribution of samples based on the first two principal coordinates (PC_o_) provided by their genomic distance matrix. The variance explained by each PC_o_ is indicated within brackets. **b** and **c** The results of admixture analyses with inferred numbers of clusters i.e. K = 2 (**b**) and K = 3 (**c**). White dashed lines separate the experimental lines. Each sample is represented by a vertical bar partitioned into stained segments according to its proportion of ancestry in each of the clusters

The first two principal coordinates obtained from multidimensional scaling (MDS) accounted for 7.88% of the total genomic variance in the subsample used in this study. PC_O_1 completely separates NeC from NeS and NeT, while PC_O_2 partially separates the NeS and NeT lines (Fig. 2a). The observed variation within each cluster was roughly proportional to its size, although the relatively higher diversity in NeT may be due to the gene flow that occurred in this line. The pattern of dispersion with the two selection lines being separated from NeC suggests that selection might be a significant force leading to genomic differentiation between these lines. However, since it is known that larger numbers of sires were transferred from NeS to NeT than from NeC to NeT, which results in NeS and NeT being more genetically connected, this might also have contributed to the observed pattern.

We performed an admixture analysis to estimate the proportions of ancestry in NeC, NeS and NeT with various numbers of clusters (K), which represent hypothetical ancestral populations. When K = 2, the clustering algorithm clearly separated NeC from NeS and NeT (Fig. 2b), and the gene flow into NeT became evident with K = 3 (Fig. 2c). Both MDS and admixture analyses identified NeT animals that showed a high degree of shared ancestry with the NeS cluster (Fig. 2a, c), which is likely due to the possible use of NeS sires for reproduction in the NeT line.

*F*_ST_ is a widely used metric for identifying population differentiation with values ranging from 0 to 1, where values close to 0 indicate more homogeneous populations and values close to 1 indicate more genetically diverged populations. The estimated average *F*_ST_ values in cross-line comparisons were equal to 0.05 ± 0.04 for “NeC vs. NeS”, 0.06 ± 0.04 for “NeC vs. NeT” and 0.02 ± 0.02 for “NeS vs. NeT”. These results reflect only subtle differentiation on a genome-wide scale, even after 40 years of intensive directional selection, which is consistent with the results of Flori et al. [4], who found minor genomic divergence between evolving lines of a single bovine breed. In spite of the low *F*_ST_ averages, their ranking was consistent with the population structure, with higher values being observed between NeC and one of the lines undergoing directional selection than between the two selection lines.

### Signatures of selection

**Fig. 3:**
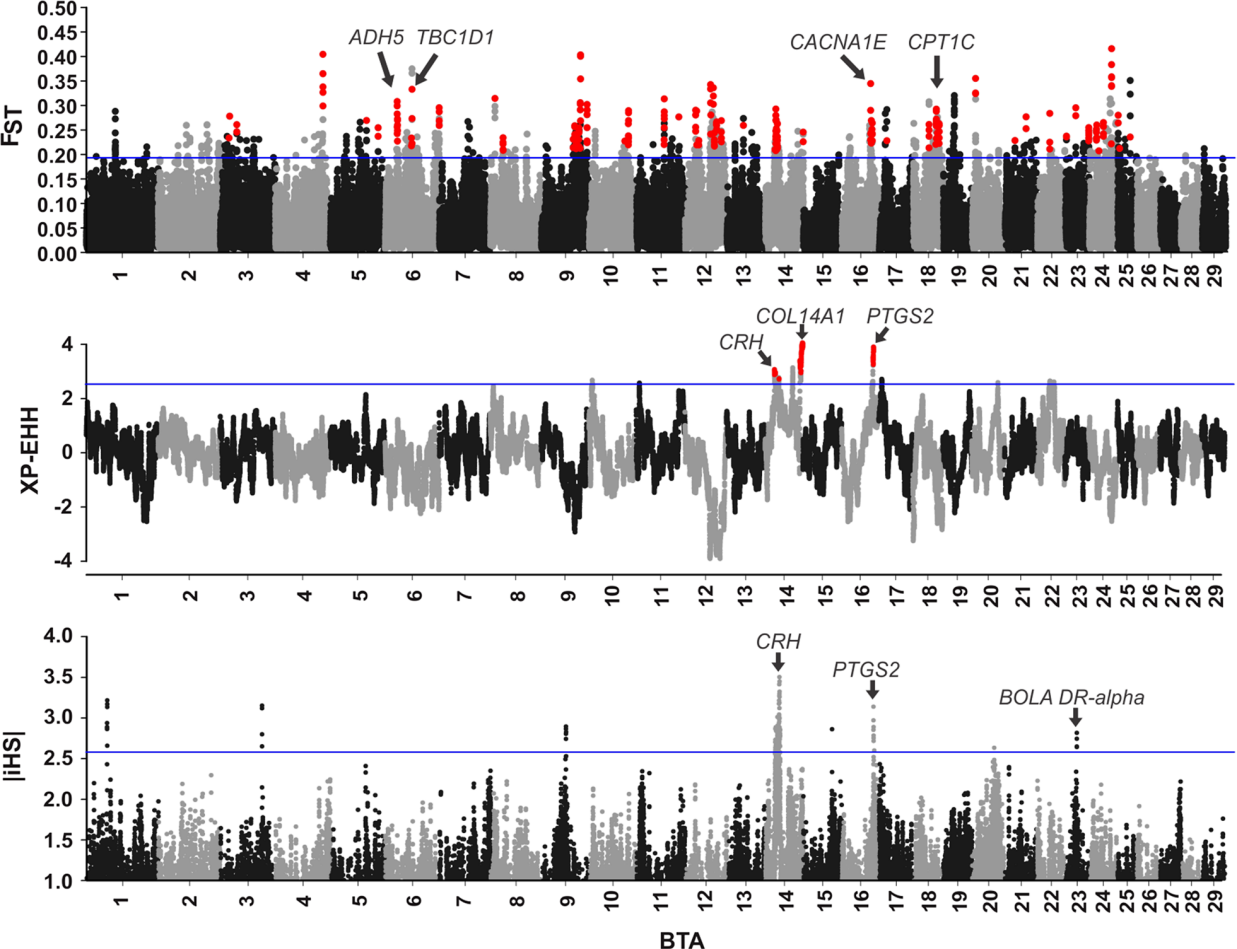
Genome-wide *F*_ST_, XP-EHH and iHS scores averaged for 100-kb windows (75 kb overlapped). Blue lines indicate the significance threshold adopted for each test. The *F*_ST_ and XP-EHH values represent the means for the same windows in the “NeC vs. NeS” and “NeC vs. NeT” comparisons. Red dots represent windows that surpass the upper-cutoff windows in both comparisons, hence putative signatures of selection

The *F*_ST_ test revealed windows with significant levels of differentiation on most of the bovine chromosomes, while XP-EHH and iHS only recognized signals on chromosomes 14 and 16 and on chromosomes 1, 3, 9, 14, 16 and 23, respectively (Fig. 3). Unusually negative XP-EHH scores, as observed on chromosome 12 (Fig. 3), may represent signatures of selection in the NeC line. However, we did not treat such scores as a signature of selection because stabilizing selection is expected to maintain haplotype diversity, rather than reduce it [39], thus producing subtle signatures of selection that cannot be detected by the XP-EHH test. Therefore, the low diversity in these regions is presumably due to genetic drift caused by the small N_e_ of the NeC line.

After combining adjacent windows that displayed signatures of selection, 48, 7 and 17 genomic regions were identified by the *F*_ST_, XP-EHH and iHS tests, respectively (see the full lists in Tables S3 and S4 [see Additional file 1: Tables S3 and S4]). In agreement with previous studies, most of the signals were detected by only one of the three approaches [40, 41]. Only six genomic regions were identified as containing signatures of selection based on two of the three metrics (Table 3).

**Table 3:** Reported QTL and genes overlapping the signatures selection that were detected by the FST, XP-EHH and iHS tests in Nelore cattle

BTA	Chromosome position (kb)	Tests	Candidate genes^1^	QTL
14	23,650–23,850	F_ST_ and iHS	*MRPL15*	Birth, weaning and yearling body weight [42]
14	24,750–24,875	F_ST_ and iHS	*TGS1*	Birth, weaning and yearling body weight [42]; meat tenderness [43]; serum IGF-1 level [44]; intramuscular fat deposition [45]
14	32,150–32,275	XP-EHH and iHS	*CRH*	Back fat thickness [46]; carcass weight [47, 48], weaning body weight [48]
14	83,850–84,000	F_ST_ and XP-EHH	*COL14A1*	Maintenance efficiency and partial efficiency of growth [49]
16	69,225–69,375	XP-EHH and iHS	*PTGS2*	Calving ease [42]
16	69,400–69,575	XP-EHH and iHS	*PLA2G4A*	Calving ease [42]

BTA, *Bos taurus* autosomes

^1^Only the best candidate genes are shown

**Fig. 4:**
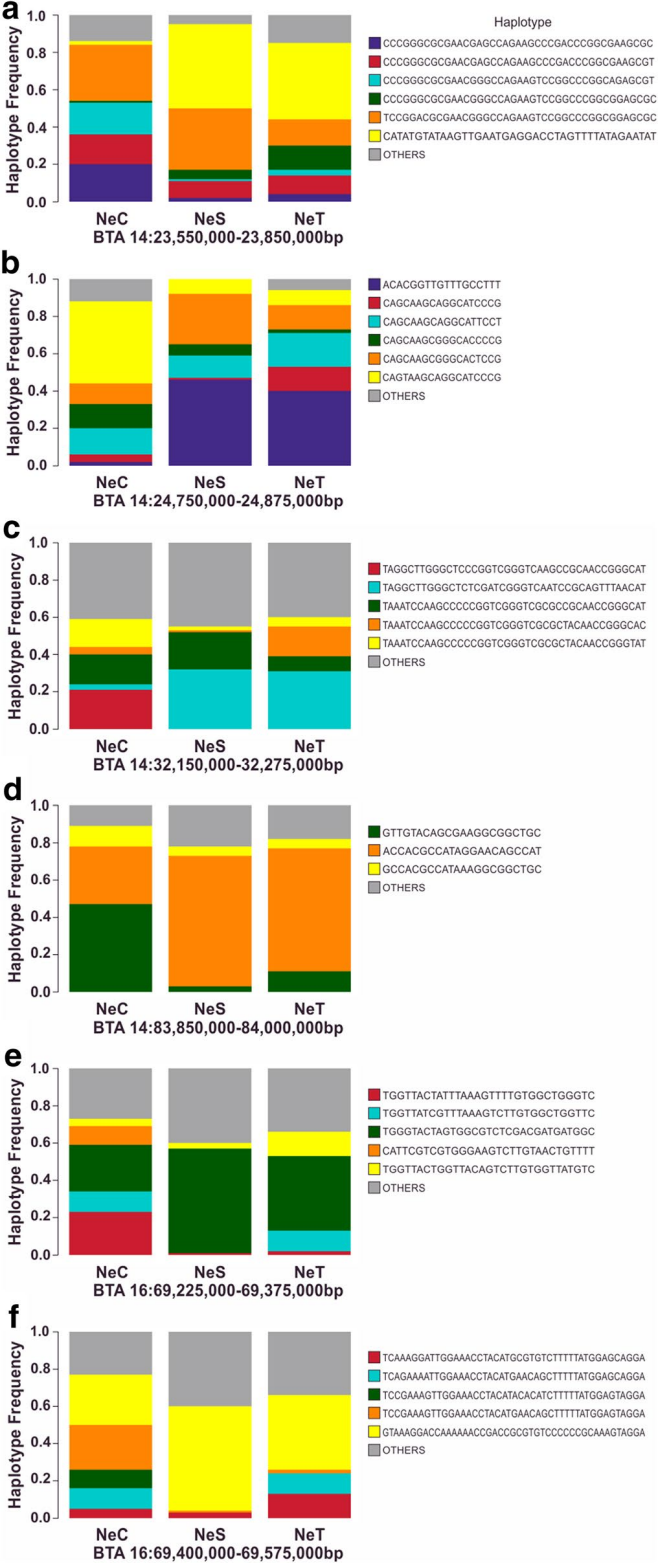
Haplotype frequencies for the four signatures of selection detected by two methods. Genomic regions were detected by both *F*_ST_ and iHS (**a**, **b**), XP-EHH and iHS (**c**, **e**, **f**) or both F_ST_ and XP-EHH (**d**). Different colors, except the grey color, refer to haplotypes with a frequency higher than 0.1 in at least one of the three lines

For the signatures of selection that were identified based on two metrics, an identical extended haplotype had the highest frequency in the lines maintained under directional selection (Fig. 4), which suggests that selection for increased YBW improved the frequencies of those haplotypes in Nelore populations, thus shaping a special pattern in these regions. The signature of selection on BTA14:83,850,000–84,000,000, which was identified by both cross-line comparison metrics (*F*_ST_ and XP-EHH), overlaps with a QTL that affects efficiency of metabolizable energy utilization for maintenance and growth, as previously observed in Nelore cattle [49]. The *COL14A1* (*collagen type XIV alpha 1 chain*) gene present in this region encodes a collagen isoform that plays a role in muscle development [50]. The signature of selection on BTA14:32,150,000–32,275,000, identified by both iHS and XP-EHH, overlaps with a QTL that affects several growth traits in beef breeds [47, 48, 51]. The most interesting candidate gene residing in this region, *CRH* (*corticotropin releasing hormone*), regulates appetite and controls the release of growth inhibitors in various organisms [51, 52]. Two additional candidate signatures of selection, detected via iHS and XP-EHH, co-localized on chromosome 16, and overlapped with a QTL reported to affect calving ease [42].

**Fig. 5:**
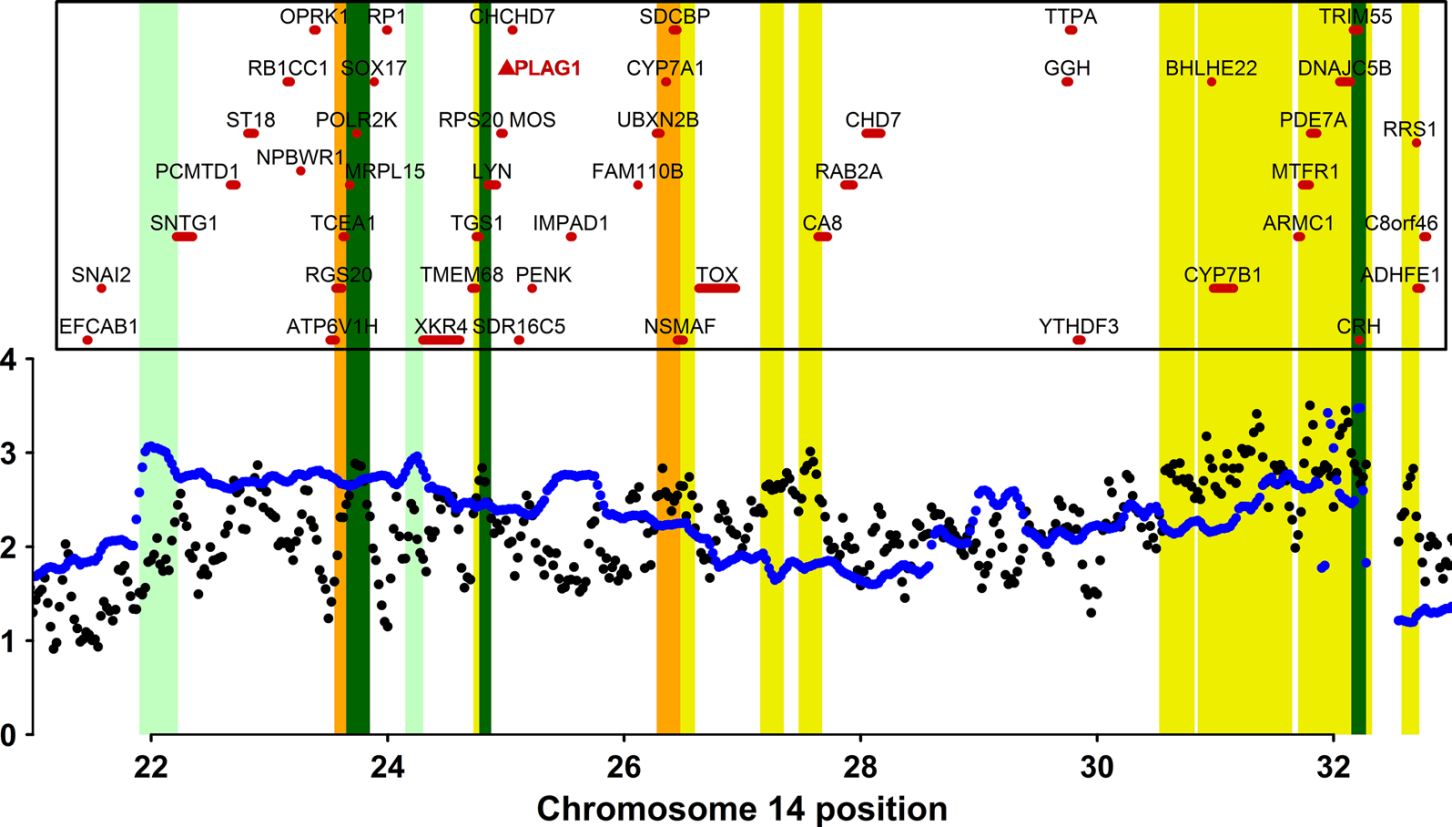
Graphical representation of signatures of selection detected via XP-EHH and iHS within a hotspot region on chromosome 14. Grey and blue represent XP-EHH and iHS scores, respectively, for 100-kb windows; light green, orange and yellow boxes indicate signals detected via iHS, *F*_ST_ and XP-EHH, respectively. The dark green boxes indicate overlap between two tests

In addition to the two coincident signals detected by both iHS and *F*_ST_ and the signal identified by both iHS and XP-EHH, within the 21–32 Mb region on chromosome 14, several other narrow regions were identified exclusively based on one of the tests (Fig. 5). In beef breeds, several studies have reported signatures of selection within this genomic interval, which are mainly in close vicinity to the *PLAG1* gene (BTA14:25,007,291–25,009,296) [5, 53, 54], which is one of the major genes that affect bovine stature and other growth traits [55]. This genomic segment also includes QTL that were previously detected in Nelore cattle and affect sexual precocity, meat quality, body and carcass weight [43, 56–58]. Fortes et al. [44] reported a significant association between several SNPs residing in this region and IGF1 serum levels in Brahman cattle. The segregation of this QTL in the Nelore breed could explain the signatures of selection that were detected in the lines undergoing directional selection for increased YBW, since IGF1 is a growth stimulator.

Some interesting genomic regions were detected exclusively based on one of the three metrics. For instance, the signature of selection on BTA6:58,700,000–58,925,000 that was revealed by the *F*_ST_ test overlaps with a previously reported signature of selection in Limousin cattle [5]. The candidate gene located in this region, *TBC1D1* (*TBC1 domain family member 1*), encodes a well-known body weight regulator in several species, including humans, mice, chickens and pigs [59–63], that is directly related to insulin-stimulated glucose uptake in muscle cells [64]. Another candidate signature of selection identified via *F*_ST_, BTA6:118,650,000–118,800,000, is linked to a QTL for average daily gain that was previously detected in the same population as that used here [16]. This signature of selection could reflect the influence of the selection for YBW on the daily rate of weight gain. This region includes the *PSAPL1* (*prosaposin*-*like 1*) gene and coincides with a copy number variant (CNV) region reported in Nelore cattle [65]. Previous studies have indicated a high level of coincidence between signatures of selection and CNV regions in cattle, which suggests that CNV may have a role in recent selection [66, 67].

Three of the signatures of selection detected via XP-EHH in our study (BTA14:77,975,000–78,875,000, BTA14:80,525,000–84,675,000 and BTA16:69,100,000–70,900,000) correspond to signals of recent positive selection that were previously reported in Nelore cattle [68]. In addition, the *XKR4* (*XK related 4*) gene resides in the signature of selection on BTA14:24,150,000–24,300,000, which is a well-characterized gene associated with growth traits in cattle, including Nelore birth weight and meat tenderness [43, 57].

In addition to the signatures of selection that could be associated with directional selection for growth, the iHS test revealed signatures of selection that represent historical selection for attributes that have become fixed in the Nelore breed. For instance, the signature of selection on BTA23:25,450,000–25,625,000 overlaps with the *Bola DR*-*alpha* gene, which is involved in the bovine immune system and may be related to adaptation and high resistance to parasites. In addition, the signature of selection on BTA14:30,850,000–31,650,000 coincides with a QTL that was reported in the Nelore breed [69] and affects alignment and appropriate angles of feet and legs, which are phenotypes that have been strongly selected for in animals registered with the Brazilian breed association ever since the breed was first established in the country.

## Conclusions

We describe a genomic scan in an indicine breed that has been selected for growth traits and is crucial for beef production in tropical environments. Our findings demonstrate that genetic stratification has occurred in this experimental Nelore population due to almost four decades of divergent selection. Three complementary approaches for the detection of signatures of selection were used to identify candidate regions on the autosomes, most of which overlapped with or were near known QTL and candidate genes that affect essential economic traits of beef cattle. The panel of identified loci includes well-known genes related to growth traits, such as *TBC1D1*, *CRH*, and *XKR4*, as well as novel candidates, such as *COL14A1*, *PSAPL1* and *CPT1C1*. The candidate signatures of selection and the list of candidate genes presented here provide a basis to better understand the metabolic mechanisms that underlie the growth traits, which have been modified by selection for YBW. Moreover, these results provide candidate genes for further fine-mapping studies.

## Additional files

**Additional file 1: Table S1.** Distribution of genotyped animals by line and generation. **Table S2.** Distribution of SNPs after quality control. **Table S3.** Genomic regions presenting extreme *F*_ST_ values with candidate genes. **Table S4.** Genomic regions presenting extreme XP-EHH and |iHS| values with candidate genes.

**Additional file 2: Figure S1.** Decay of r^2^ as a function of physical distance on chromosomes 11 and 12. **Figure S2.** Genome-wide *F*_ST_ scores in overlapping windows for the “NeC vs. NeS” (upper) and “NeC vs. NeT” (bottom) comparisons. **Figure S3.** Genome-wide XP-EHH scores in overlapping windows for the “NeC vs. NeS” (upper) and “NeC vs. NeT” (bottom) comparisons. **Figure S4.** Correlation between SNP iHS values when considering ancestral allele definitions from available lists (|iHS|_1) and randomly defining one allele as ancestral (|iHS|_2). **Figure S5.** Minor allele frequency distributions in three selection lines.
