# Supplementary material for: Genome-wide scan reveals population stratification and footprints of recent selection in Nelore cattle
Source: Genet Sel Evol. 2018 May 2;50:22. doi: 10.1186/s12711-018-0381-2 (PMC5930444; doi:10.1186/s12711-018-0381-2)
Supplement: Supplementary file 1 — Additional file 1: Table S1. Distribution of genotyped animals by line and generation. Table S2. Distribution of SNPs after quality control. Table S3. Genomic regions presenting extreme FST values with candidate genes. Table S4. Genomic regions presenting extreme XP-EHH and |iHS| values with candidate genes. [file 12711_2018_381_MOESM1_ESM.docx]

**Table S1. Distribution of genotyped animals by line and generation.**

| Generation^1^ | Lines | | | | | | | | | |  | Total | |
| --- | --- | --- | --- | --- | --- | --- | --- | --- | --- | --- | --- | --- | --- |
|  | NeC | | |  | NeS | | |  | NeT | |  |  | |
|  | Males | Females | | Males | | Females | | Males | | Females | Males | | Females |
| 4 | 2 | | 3 | 2 | | | 11 | 11 | | 9 | 15 | | 23 |
| 5 | 14 | | 18 | 30 | | | 52 | 87 | | 88 | 131 | | 158 |
| 6 | 30 | | 4 | 67 | | | - | 159 | | 111 | 256 | | 115 |
| 7 | 18 | | - | 27 | | | - | 16 | | 4 | 61 | | 4 |
| Total | 64 | | 25 | 126 | | | 63 | 273 | | 212 | 463 | | 300 |

NeC: Line Nellore Control ; NeS: Line Nellore Selection ; NeT: Line Nellore Traditional

^1^ Individual Generation Coefficients were truncated to categorise samples in classes

**Table S2. Distribution of SNPs after quality control.**

| BTA | Size^1^  (Mb) | SNP  (N) | Mean distance ± SD (Kb) | Min. distance  (Kb) | Max. distance  (Kb) |  |
| --- | --- | --- | --- | --- | --- | --- |
| 1 | 158.3 | 33,773 | 4.7 ± 5.3 | 0.066 | 173.7 | |
| 2 | 137.1 | 28,627 | 4.8 ± 5.3 | 0.021 | 137.1 | |
| 3 | 121.4 | 25,517 | 4.8 ± 6.0 | 0.020 | 224.4 | |
| 4 | 120.8 | 25,401 | 4.7 ± 5.5 | 0.066 | 174.9 | |
| 5 | 121.2 | 23,986 | 5.0 ± 6.9 | 0.021 | 299.5 | |
| 6 | 119.4 | 26,804 | 4.5 ± 6.8 | 0.031 | 613.9 | |
| 7 | 112.6 | 23,905 | 4.7 ± 9.7 | 0.003 | 1080.0 | |
| 8 | 113.4 | 24,324 | 4.7 ± 6.8 | 0.003 | 423.9 | |
| 9 | 105.7 | 24,470 | 4.3 ± 4.5 | 0.042 | 95.83 | |
| 10 | 104.3 | 21,526 | 4.8 ± 7.1 | 0.028 | 338.2 | |
| 11 | 107.3 | 22,435 | 4.8 ± 5.8 | 0.023 | 121.8 | |
| 12 | 91.2 | 18,055 | 5.0 ± 13.2 | 0.029 | 880.0 | |
| 13 | 84.2 | 16,466 | 5.1 ± 6.4 | 0.034 | 261.7 | |
| 14 | 84.6 | 19,488 | 4.3 ± 6.9 | 0.091 | 454.0 | |
| 15 | 85.3 | 18,241 | 4.7 ± 6.3 | 0.056 | 244.3 | |
| 16 | 81.7 | 17,539 | 4.7 ± 7.5 | 0.037 | 443.5 | |
| 17 | 75.2 | 16,174 | 4.7 ± 8.1 | 0.036 | 504.8 | |
| 18 | 66.0 | 14,030 | 4.7 ± 6.9 | 0.087 | 187.8 | |
| 19 | 64.0 | 12,214 | 5.2 ± 7.5 | 0.152 | 161.1 | |
| 20 | 72.0 | 15,487 | 4.6 ± 5.4 | 0.021 | 92.79 | |
| 21 | 71.6 | 15,271 | 4.7 ± 5.9 | 0.047 | 148.4 | |
| 22 | 61.4 | 12,701 | 4.8 ± 7.2 | 0.062 | 361.7 | |
| 23 | 52.5 | 10,868 | 4.8 ± 7.7 | 0.026 | 406.8 | |
| 24 | 62.7 | 12,789 | 4.9 ± 6.1 | 0.035 | 139.8 | |
| 25 | 42.9 | 8,321 | 5.1 ± 6.7 | 0.153 | 125.6 | |
| 26 | 51.7 | 11,535 | 4.5 ± 5.8 | 0.153 | 181.2 | |
| 27 | 45.4 | 9,696 | 4.7± 11.4 | 0.163 | 842.1 | |
| 28 | 46.3 | 9,170 | 5.0 ± 7.2 | 0.023 | 194.6 | |
| 29 | 51.5 | 10,359 | 5.0 ± 7.8 | 0.125 | 361.6 | |
| TOTAL | 2,511.7 | 529,172 |  |  |  |  |
| Mean |  |  | 4.7 ± 7.1 | 0.003 | 1080.0 |  |

BTA: Bos taurus chromosome;

^1^ Autosomal chromosomes lengths based on UMD 3.1.1

**Table S3. Genomic regions representing extreme *F_ST_* with candidate genes.**

| Genomic Region^1^* | Candidate Genes |
| --- | --- |
| BTA3:35,975-36,150 | *NTNG1* |
| BTA4:104,275-104,500 | *TBXAS1, PARP12, KDM7A* |
| BTA5:105,200-105,325 | *NTF3* |
| BTA6:26,050-26,275 | *H2AFZ, DNAJB14, LAMTOR3, DAPP1* |
| BTA6:26,725-26,950 | *ADH6, ADH4, ADH5, METAP1* |
| BTA6:57,450-57,600 |  |
| BTA6:58,775-58,925 | *PGM2, TBC1D1* |
| BTA6:118,625-118,775 | *PSAPL1* |
| BTA8:27,050-27,350 |  |
| BTA9:73,225-73,450 | *SGK1* |
| BTA9:76,275-76,425 | *OLIG3* |
| BTA9:82,475-82,675 | *STX11, SF3B5* |
| BTA9:85,450-85,600 |  |
| BTA9:86,025-86,200 |  |
| BTA9:100,325-100,575 |  |
| BTA10:85,550-85,725 | *PTGR2, ZNF410, FAM161B* |
| BTA10:85,850-86,175 | *LIN52, VSX2, ABCD4, VRTN, SYNDIG1L, NPC2* |
| BTA11:60,250-60,375 |  |
| BTA11:60,500-60,675 | *FAM161A, COMMD1, B3GNT2* |
| BTA12:22,325-22,500 |  |
| BTA12:24,625-24,800 | *SUPT20H*, *EXOSC8*, *ALG5*, *SMAD9* |
| BTA12:29,150-29,350 | *RXFP2* |
| BTA12:55,200-55,375 |  |
| BTA12:63,525-63,775 |  |
| BTA12:68,500-68,675 | *GPC6* |
| BTA12:78,950-79,125 | *IPO5* |
| BTA14:23,550-23,850 | *ATP6V1H, RGS20, TCEA1, LYPLA1, MRPL15, POLR2K* |
| BTA14:24,750-24,875 | *TGS1, LYN* |
| BTA14:26,275-26,475 | *UBXN2B, CYP7A1, SDCBP, NSMAF* |
| BTA14:83,875-84,000 | *COL14A1* |
| BTA16:63,975-64,125 | *CACNA1E* |
| BTA16:66,100-66,275 | *NCF2, ARPC5, APOBEC4* |
| BTA18:34,175-34,400 | *BEAN1, TK2* |
| BTA18:50,175-50,500 | *LTBP4, NUMBL, SNRPA, ITPKC* |
| BTA18:52,450-52,575 | *ZNF227, ZNF235* |
| BTA18:56,500-56,800 | *CPT1C, TSKS, MED25, PTOV1, PNKP, AKT1S1, TBC1D17, IL4I1, ATF5, VRK3* |
| BTA20:6,650-6,800 | *FAM169A, NSA2, GFM2* |
| BTA21:45,875-46,000 | *PSMA6* |
| BTA22:26,475-26,625 |  |

**… Continuation Table S3**

| Genomic Region^1^* | Candidate Genes |
| --- | --- |
| BTA23:1,875-2,000 |  |
| BTA23:22,350-22,475 |  |
| BTA23:50,875-51,150 |  |
| BTA24:15,600-15,800 |  |
| BTA24:16,325-16,450 |  |
| BTA24:31,025-31,200 | *SS18* |
| BTA24:48,900-49,150 | *CTIF, SMAD7* |
| BTA25:900-1,050 | *CACNA1H, UBE2I* |

^1^Chromosome:StartPosition(kb):EndPosition(kb );

*Only regions composed by two or more consecutive 100Kb windows are presented.

**Table S4. Genomic regions representing extreme XP-EHH and |iHS| with candidate genes**

| Genomic Region ^1^* | Test | Candidate Genes |
| --- | --- | --- |
| BTA1:44,850-45,150 | iHS | *KAT2B, SGO1, ZNF96* |
| BTA3:91,100-91,275 | iHS | *-* |
| BTA9:54,025-54,225 | iHS | *FHL5, UFL1* |
| BTA14:21,900-22,225 | XP-EHH | *SNTG1* |
| BTA14:23,650-23,850 | iHS | *MRPL15, POLR2K* |
| BTA14:24,150-24,300 | XP-EHH | *XKR4* |
| BTA14:24,725-24,875 | iHS | *TMEM68, TGS1, LYN* |
| BTA14:26,425-26,600 | iHS | *SDCBP, NSMAF* |
| BTA14:27,150-27,350 | iHS | *CA8* |
| BTA14:27,475-27,675 | iHS | *-* |
| BTA14:30,525-30,825 | iHS | *-* |
| BTA14:30,850-31,650 | iHS | *CYP7B1, BHLHE22* |
| BTA14:31,700-32,325 | iHS | *ARMC1, MTFR1, PDE7A, DNAJC5B, RRS1, TRIM55, CRH* |
| BTA14:32,150-32,275 | XP-EHH | *TRIM55, CRH* |
| BTA14:32,575-32,725 | iHS | *ADHFE1* |
| BTA14:33,225-33,375 | iHS | *COPS5, CSPP1, ARFGEF1* |
| BTA14:33,625-33,800 | iHS | *CPA6* |
| BTA14:77,975-78,875 | XP-EHH | *CNGB3,* *CPNE3,* *RMDN1,* *WWP1* |
| BTA14:80,525-84,675 | XP-EHH | *SNX16, CHMP4C, ZFAND1, IMPA1, ENPP2, TAF2, DSCC1, DEPTOR, COL14A1, MRPL13, MTBP, SNTB1, SLC10A5* |
| BTA16:69,100-70,900 | XP-EHH | *PTGS2, PLA2G4A, KCNK2, CENPF,* *PTPN14, SMYD2, RNPEP, ELF3, GPR37L1,* *ARL8A* |
| BTA16:69,225-69,375 | iHS | *PTGS2* |
| BTA16:69,400-69,575 | iHS | *PLA2G4A* |
| BTA23:25,450-25,625 | iHS | *Bola DR-alpha* |

^1^Chromosome:StartPosition(kb):EndPosition(kb);

*Only regions composed by two or more consecutive 100Kb windows are presented.
