# Supplementary material for: Genome-wide scan reveals population stratification and footprints of recent selection in Nelore cattle
Source: Genet Sel Evol. 2018 May 2;50:22. doi: 10.1186/s12711-018-0381-2 (PMC5930444; doi:10.1186/s12711-018-0381-2)
Supplement: Supplementary file 2 — Additional file 2: Figure S1. Decay of r2 as a function of physical distance on chromosomes 11 and 12. Figure S2. Genome-wide FST scores in overlapping windows for the “NeC vs. NeS” (upper) and “NeC vs. NeT” (bottom) comparisons. Figure S3. Genome-wide XP-EHH scores in overlapping windows for the “NeC vs. NeS” (upper) and “NeC vs. NeT” (bottom) comparisons. Figure S4. Correlation between SNP iHS values when considering ancestral allele definitions from available lists (|iHS|_1) and randomly defining one allele as ancestral (|iHS|_2). Figure S5. Minor allele frequency distributions in three selection lines. [file 12711_2018_381_MOESM2_ESM.docx]

**
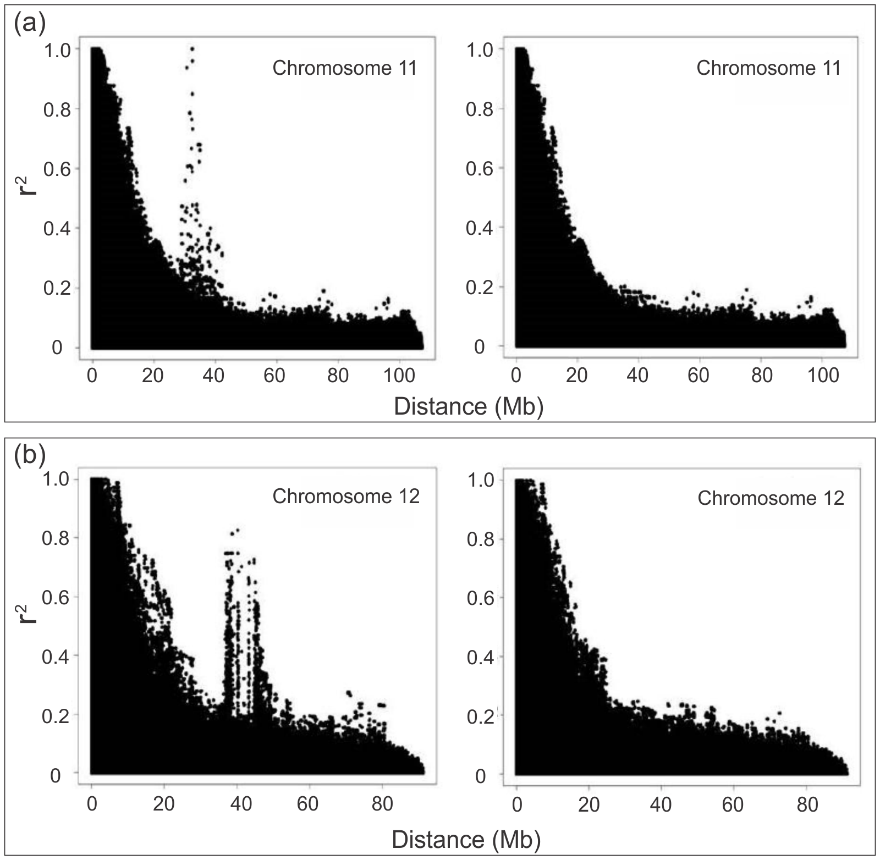
**

**Figure S1. Decay of r^2^ as a function of physical distance on chromosomes 11 and 12.** Panels (a) and (b) represent the pairwise LD before (left) and after (right) removal of 1 and 154 possible misplaced SNPs on chromosomes 11 and 12, respectively.

**
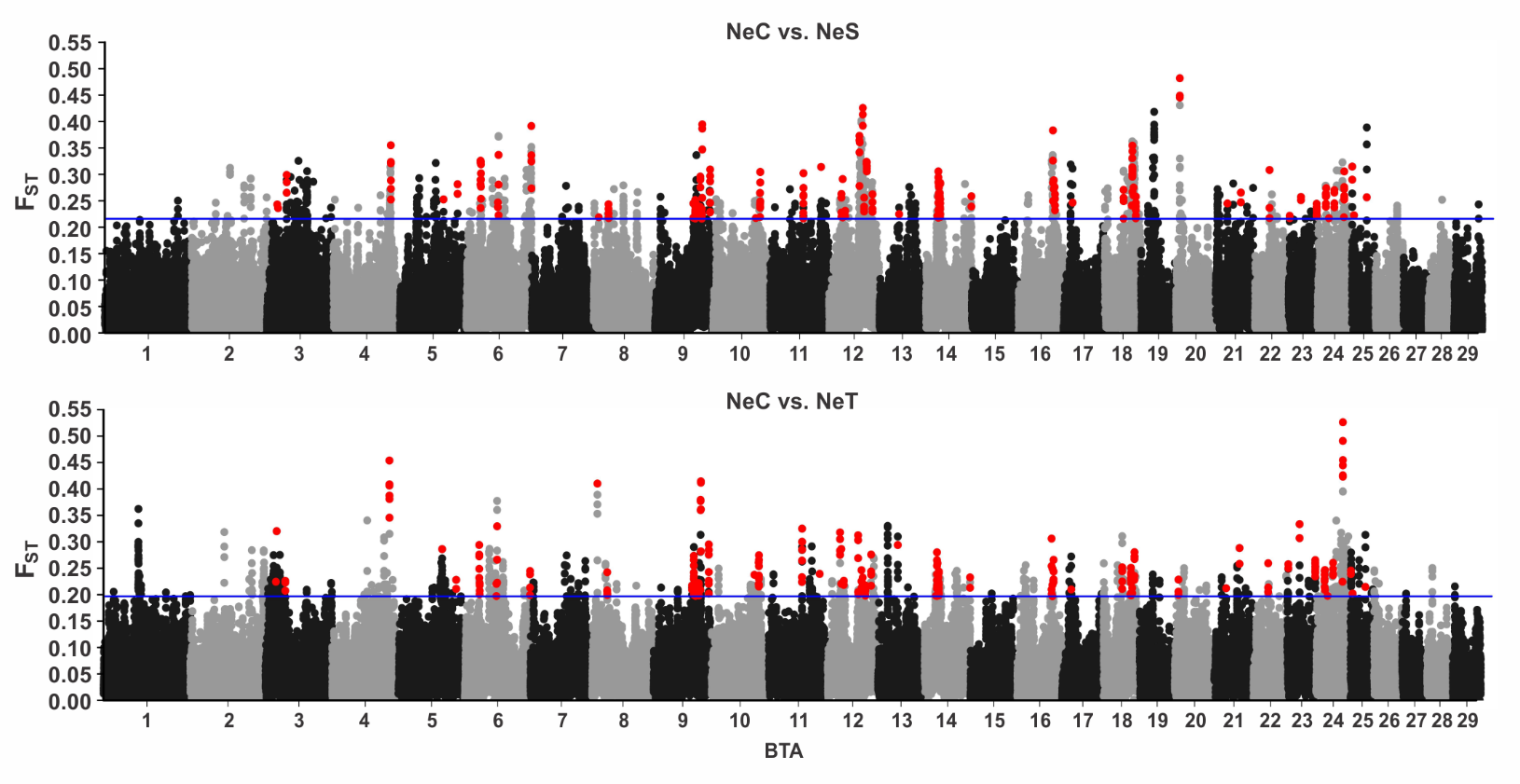
**

**Figure S2. Genome-wide *F*_ST_ scores in overlapping windows for the “NeC vs. NeS” (upper) and “NeC vs. NeT” (bottom) comparisons.** Horizontal blue lines indicates the top 1% of the F_ST_ distribution under each comparison scheme. Red dots indicate windows exceeding the 1% threshold and outside of the 95% confidence interval of their heterozygosity bin in both comparisons.


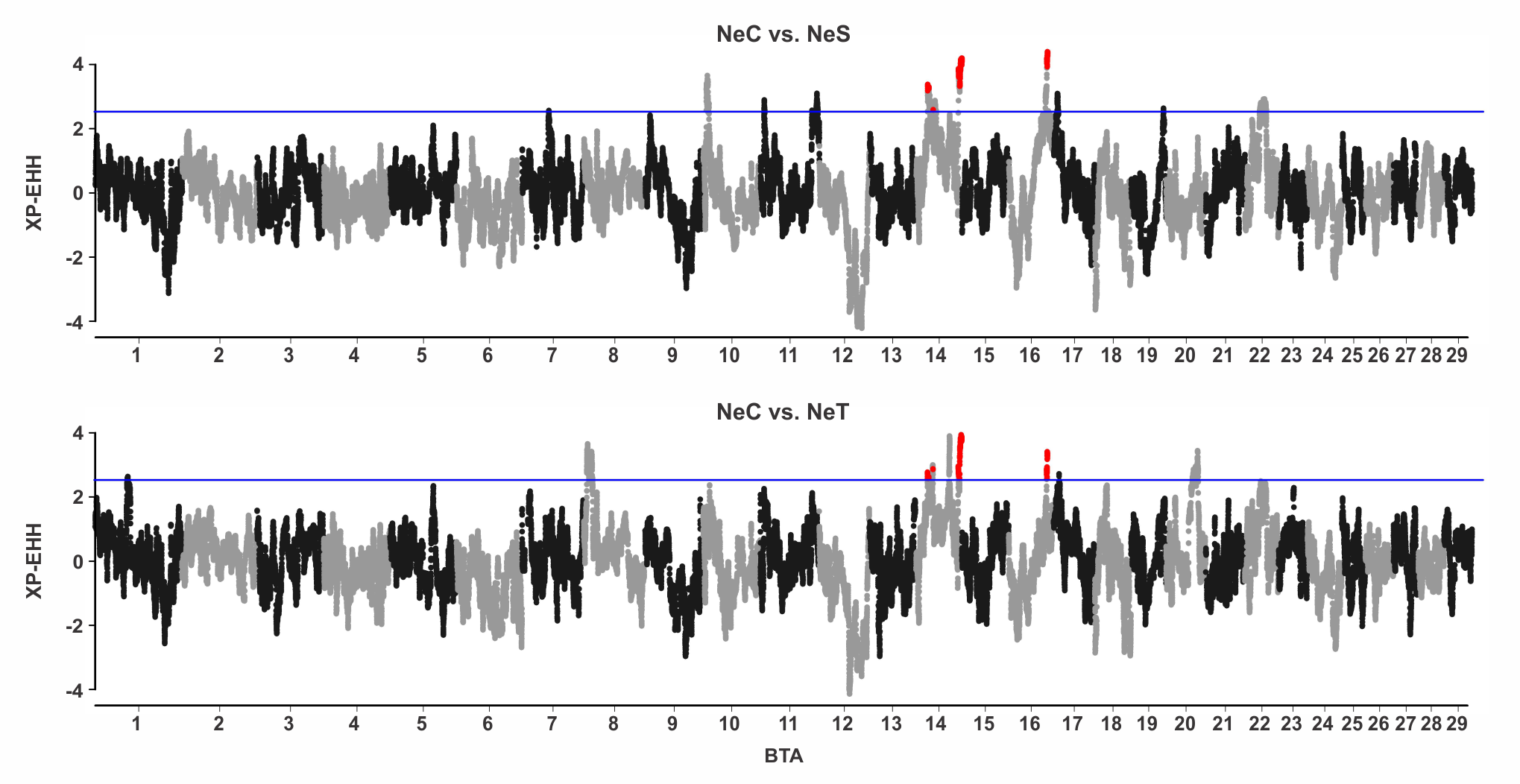


**Figure S3.** **Genome-wide XP-EHH scores in overlapping windows for the “NeC vs. NeS” (upper) and “NeC vs. NeT” (bottom) comparisons.** Red dots indicate the windows above the upper threshold identified in both comparisons.


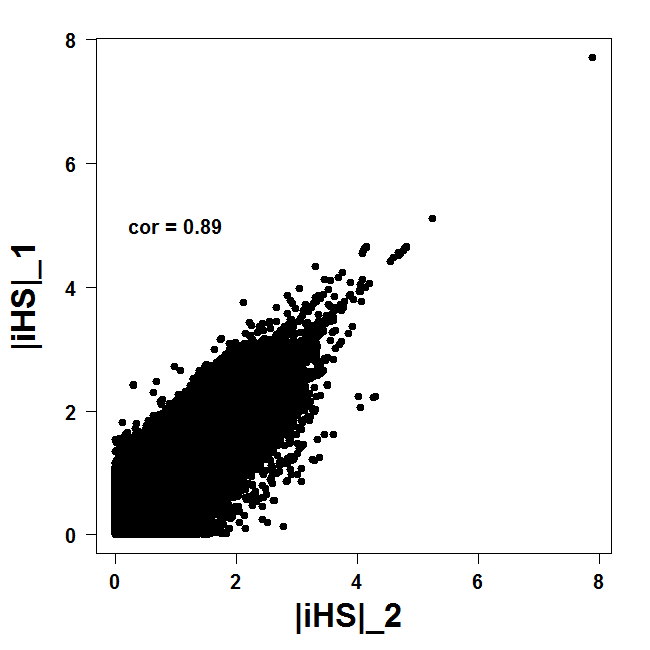


**Figure S4. Correlation between SNP iHS values when considering ancestral allele definitions from available lists (|iHS|_1) and randomly defining one allele as ancestral (|iHS|_2).**

**
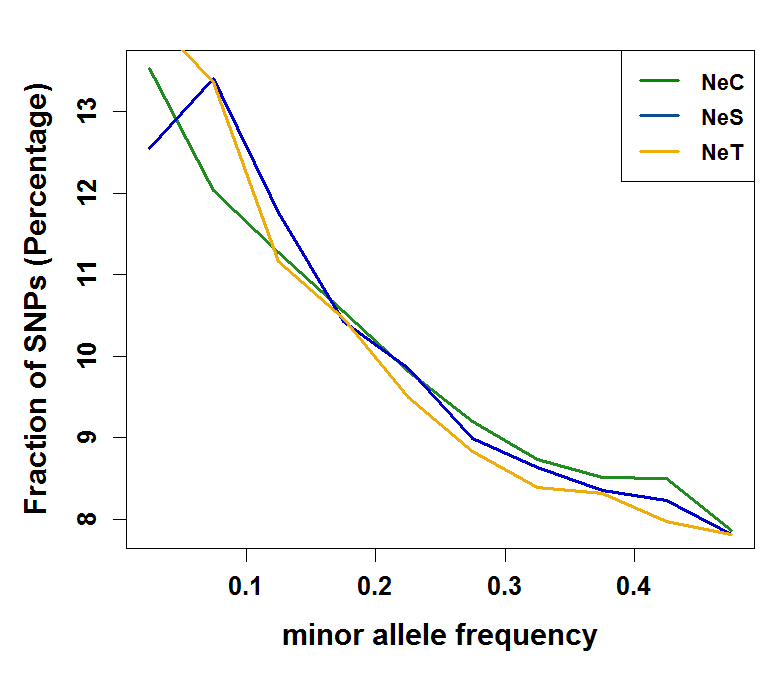
**

**Figure S5.** Minor allele frequency distributions in three selection lines
